# Establishment of Normal Reference Intervals for CD3^+^, CD4^+^, CD8^+^, and CD4^+^ to CD8^+^ Ratio of T Lymphocytes in HIV Negative Adults from University of Gondar Hospital, North West Ethiopia

**DOI:** 10.1155/2014/267450

**Published:** 2014-11-18

**Authors:** Addisu Gize, Biniam Mathewos, Beyene Moges, Meseret Workineh, Lealem Gedefaw

**Affiliations:** ^1^Department of Medical Laboratory Science and Pathology, Jimma University, Jimma, Ethiopia; ^2^Department of Immunology and Molecular Biology, College of Medicine and Health Sciences, University of Gondar, Ethiopia

## Abstract

*Background.* Reference values for the CD3^+^, CD4^+^, CD8^+^, and CD4^+^ to CD8^+^ ratio T lymphocyte subsets are adopted from textbooks. But for appropriate diagnosis, treatment, and follow-up of patients, correct interpretations of the laboratory results from normal reference interval are mandatory. This study was, therefore, planned to establish normal reference interval for T lymphocytes subset count and CD4^+^ to CD8^+^ ratio. *Methods.* A cross-sectional study was conducted on apparently healthy adult individuals who visited voluntary counseling and HIV testing clinic Gondar University Hospital from April to May, 2013. Whole blood was analyzed using fluorescence-activated cell sorting (BD FACS, San Jose, CA) machine to enumerate the T-cell subpopulations. *Results.* Out of the total 320 study participants, 161 (50.3%) were men and 159 (49.7%) were women. The normal reference intervals were (655–2,823 cells/*μ*L), (321–1,389 cells/*μ*L), and (220–1,664 cells/*μ*L) for CD3^+^, CD4^+^, and CD8^+^ T lymphocyte subsets, respectively, and CD4^+^ to CD8^+^ ratio was 0.5–2.5. *Conclusion.* The overall CD3^+^ T lymphocytes reference interval in the current study was wide; low CD4^+^ T lymphocytes, CD4 to CD8 ratio, and high CD8^+^ T lymphocytes values were observed.

## 1. Introduction

T lymphocyte cells are defined by the expression of CD3^+^ molecules. The most important cell-surface molecules for identifying T lymphocyte subpopulations have been CD4^+^, CD8^+^, and T-cell receptor complex molecules (CD3^+^, the T-cell receptor *α* and *β* chains [1].

Those cells which express CD4^+^ and CXCR4 cell-surface molecules serve as a receptor and coreceptor for human immunodeficiency virus (HIV), respectively. HIV infects CD4^+^ T lymphocytes mainly, replicates within them, and lyses the cells as the replicated virions are released extracellularly to infect yet other CD4^+^ cells [2].

In advanced disease, most of the observed immune defects can ultimately be explained by the quantitative depletion of CD4^+^ T lymphocytes and the severity is reflected by measures of the absolute CD4^+^ T lymphocyte numbers per blood volume, the percentages of CD4^+^ lymphocyte (CD4^+^%) among lymphocytes, and also the CD4^+^/CD8^+^ ratio [3].

CD4^+^ T lymphocyte cells coordinate the immune system's response to the pathogens [4], and hence, normal reference interval values are critically important for the correct interpretation of diagnostic tests [5]. The influence of geographical location, racial and ethnic background, age, sex, and conditions of living on the distribution of human peripheral blood T lymphocyte subpopulations has already been documented in various studies of a given population in different parts of the world [6–8].

Few studies conducted in Africa show significant reference range variations of CD4^+^ T-cell and CD8^+^ T-cells within African populations because of environmental, sociodemographic differences and because of biological influences such as age, altitude, and ethnic origin [9–12].

Ethiopia has great geographical diversity [13], but in the national context, reference values for Ethiopians have never been established, despite a very few attempts in few localities which showed variation from other countries [14].

For appropriate diagnosis, treatment, and follow-up of patients, correct interpretation of the laboratory results is mandatory since it helps clinicians. This is attained by the knowledge of normal reference intervals that have been established at local setting than using references from western countries which have different climate, socioeconomic status, living style and genetic makeup, and so forth [15]. This study is, therefore, planned to provide normal reference intervals of T lymphocytes subsets and CD4^+^ to CD8^+^ cells ratio for the population of Gondar, North West Ethiopia.

## 2. Materials and Methods

### 2.1. Study Design and Study Subjects

Cross-sectional study was conducted from April to May, 2013, at the voluntary counseling and HIV testing clinic of University of Gondar Hospital and all laboratory investigations were done in the antiretroviral therapy laboratory of the hospital, Gondar town, North West Ethiopia. This laboratory, according to Ethiopian National Accreditation Office (ENAO), which established the Stepwise Laboratory (Quality) Improvement Process towards Accreditation (SLIPTA), has got 3 stars (75–84%) ranked from 5 stars (≥95%). ENAO gives recognition based on progress towards meeting requirements set by international standards and on laboratory performance during the 12 months preceding the SLIPTA audit, relying on complete and accurate data, to improve quality of public health laboratories in developing countries to achieve ISO 15189 standards.

All study participants who were free from clinical disease conditions, looking apparently healthy, whose age groups 18 to 64 years old, who lived in the area at least in the past for six months, being negative for HIV-1/2 antibodies, and having body mass index between 16.5 Kg/M^2^ and 22 Kg/M^2^ were included. The participants giving history of the recent enteric, respiratory infections and malaria and chronic noninfectious illnesses like diabetes mellitus, chronic renal disease, allergies, recent past immunization in the last 6 months, steroid therapy in the past three months, antibiotic usage four weeks prior to enrollment, and blood or blood product transfusion in the past 6 months were excluded. Female participants who were pregnant after being confirmed by human chorionic gonadotropin (hcG) tests were also excluded from the study.

A total of 320 individuals were included based on the recommended guideline of Clinical and Laboratory Standards Institute (CLSI) [21]. Accordingly this CLSI, a priori sampling method was conducted that requires well-defined exclusion and partitioning criteria before the selection of the reference individuals.

### 2.2. Data Collection and Processing

Prior to actual data collection, the clarity of the tool was assessed using 5% of the total sample size on other study areas. In addition, training about data collection was given for data collectors, two HIV counselor nurses and three laboratory technologists. All specimens from study subjects were handled following the standard operating procedures. Possible diurnal variations for T lymphocyte counts were controlled by collecting blood only at the specified hours in the morning (between 8:30 and 10:30 a.m.). The proper functioning of instruments, laboratory reagents, and technical performance were checked daily using quality control samples before running participant samples and along with participant samples.

Using the semistructured and pretested questionnaire sociodemographic characteristics and relevant clinical data were collected by face-to-face interview from the study participants. Five (5) mL of whole blood for all HIV1/2 antibodies screening CD3^+^, CD4^+^, CD8^+^,  and CD4^+^ to CD8^+^ ratio T lymphocytes counts was collected in tripotassium ethylenediaminetetraacetic acid (K_3_EDTA) vacutainer tubes.

Participant's specimen screened for the presence of  HIV antibodies using HIV-1/2 rapid test assay (using KHB, STAT Pack, and UNIGOLD) following the manufacturer's instruction and the national test algorism. This HIV serology was carried out anonymously with all sociodemographic characteristics. Those confirmed negative for HIV1/2 antibodies women were requested to check their pregnancy by One Step Strip Style hCG Pregnancy Test. Finally, all HIV negative individuals and nonpregnant women samples were assayed for CD3^+^,  CD4^+^,  CD8^+^, and CD4 to CD8 ratio T lymphocytes count.

All samples for T lymphocytes count processed and counted within 8 hrs of collection at the ART Laboratory, according to the instructions of the manufacturer. The blood collected with BD vacutainer tubes was well mixed and 50 *μ*L of the whole blood was transferred into monoclonal antibodies reagent tubes for FACSCount analysis to enumerate the T lymphocytes.

In brief, 50 *μ*L of whole blood was mixed with monoclonal antibodies (MAbs) (5 *μ*L of each T lymphocytes reagent tubes) and incubated at room temperature for 15 min in separate tubes and was placed in the dark place. By adding of fluorescence-activated cell sorter lysing solution (50% diethylene glycol and 15% formaldehyde) (Becton Dickinson) erythrocytes were lysed. After vortexing the tubes, it was incubated in the dark at room temperature for 15 minutes. Cells were injected into a “flow cell” which is located in the optical path of a light source, analyzed, and counted using a fluorescence-activated cell sorting count machine (*Becton Dickinson* FACSCount flow cytometer); here, the cells come in contact with the laser light, which causes the fluorochrome-labeled cells to fluoresce. This fluorescent light provides the necessary information for the instrument to count the cells.

### 2.3. Data Analysis

Data were entered and cleaned first using Epi-Info version 3.5.1 and then exported to SPSS version 20 for analysis. The mean, median, and standard deviation of normal reference interval values was calculated. Reference interval was determined as 95th percentile of the ordering data. *P* values less than 0.05 were considered statistically significant.

### 2.4. Ethical Clearance and Consent

Ethical clearance was secured from the ethical review committee of the School of Biomedical and laboratory Science, University of Gondar. Official letter from University of Gondar Teaching Hospital Director office was obtained and submitted to both VCT center and antiretroviral therapy laboratory coordinator. Written informed consent was taken from each study participant. Those participants, who had HIV infection, were referred to the HIV care and treatment clinic for further management after counseling. Female participants who were reactive for hcG test were referred to antenatal clinic for better care.

## 3. Results

A total of 320 study participants were included in the study with mean age of 25.3 ± 7.7 years old. Out of the total 320 study participants, 161 (50.3%) were men and 159 (49.7%) were women. Men were older than women, with mean ages of 27 ± 8 years and 23.5 ± 6 years, respectively. The majority of the study participants were 224 (70%) single in marital status and were 95 (29.7%) students in their occupational status; concerning educational status most of participants 116 (36.3%) were 9th–12th grades and 292 (91.3%) Amhara ethnic (Table 1). The overall mean, median, and the 95% normal reference interval values for absolute CD3^+^, CD4^+^, and CD8^+^ T lymphocyte count and also for absolute CD4^+^ to CD8^+^ ratio were established for the participants (Table 2).

T lymphocyte subset values in HIV-1/2 seronegative individuals are reported to be affected by different factors like gender, age, and geographical location worldwide. In this study also as overall the age increases, there was decline in the value of CD8^+^ T lymphocytes (Table 3).

The absolute mean of CD3^+^ (1567 ± 562 cells/*μ*L) and CD4^+^ (759 ± 252 cells/*μ*L) T lymphocytes for men is statistically lower than women (1706 ± 547 cells/*μ*L) (*P* = 0.01) and (822 ± 275 cells/*μ*L) (*P* = 0.00), respectively, but there was no statistically significant difference in the mean absolute count for CD8^+^ T lymphocytes (*P* = 0.18) and CD4^+^ to CD8^+^ ratio of lymphocytes between male and female (*P* = 0.26).

## 4. Discussion

The mean and the upper limit reference interval results CD3^+^ T lymphocyte counts were higher than the user's guideline mean: 1206 and (reference interval: 688–1955) cells/*μ*L. The mean of CD3^+^ T lymphocyte count for women participants was significantly higher than from those men participants (*P* = 0.01).

The median (reference interval) of these lymphocytes in women seems slightly greater than the study conducted in other parts of Ethiopian median (reference interval). In addition, median and the upper limit of men CD3^+^ T lymphocyte of the current study show a higher result than men participants median and reference interval of their study, as studied by Kassu et al. [16], and lower from the study done in South West Ethiopia median (reference range) 1,643 (760–3,465) by Haileamlak et al. [22]; this could result from the difference of study subject since we used apparently healthy individuals whereas their study groups were clinically assessed specially in the Kassu et al. study. CD3^+^ T lymphocytes mean, median, and 95% reference interval of the current study are comparable with the study done in Kenya, median and reference interval [20].

The 95% reference interval for the absolute CD4^+^ T lymphocyte count for the participants was lower and a slight wider than the current national guideline for ART users and FACSCount user guideline (reference interval: 470–1298 cells/*μ*L) may be because of racial difference among the study subjects. Results of mean and median values for CD4^+^ T lymphocytes were greater than from the result of study done in Akaki factory worker by Tsegaye et al., Addis Ababa, Ethiopia (775 ± 225, 761 cells/*μ*L) and for the present study 95% normal reference interval (321–1389 cells/*μ*L) lower limit nearly lower than there (366–1235 cells/*μ*L) [14] could be due to geographical difference. CD4^+^ T lymphocytes count was consistent with the study done in Tanzania [10] and Botswana blood donors [23].

However, the values of CD4^+^ T lymphocytes mean, median, and 95% normal reference interval were lower than from the study done in Kenya [20], Uganda (mean: 938, reference interval: 418–2105 cells/*μ*L) [8], Central Africa Republic study done by Menard et al. (mean: 940, median: 912, reference interval: 386–1454 cells/*μ*L) [17], healthy Nigerian adults done (mean: 847, reference interval: 365–1571 cells/*μ*L) [11]; this was again maybe by different geographical location.

The absolute median count of CD4^+^ T lymphocytes for men (737 cells/*μ*L) and women (858 cells/*μ*L) was statistically different in the present study and lower than the study done by Torres et al. in the two regions of Brazil blood donors, that is, the study in Bahia/Pará men absolute count (897/768 cells/*μ*L) and for women (1032/802 cells/*μ*L) [7] maybe because of the use of apparently healthy study subjects in the current study.

The overall mean and median CD8^+^ T lymphocyte counts show similarity from the study done by Tsegaye et al. so far other parts of Ethiopia. CD8^+^ T lymphocyte counts show great difference from the FACSCount user guideline (mean: 346, reference interval: 208–796); it might be because of the large sample size used in the present study and/or racial diversity. Women mean and median of CD8^+^ T lymphocytes counts were higher than men but it is not statistically significant (*P* = 0.18).

Similarities had also observed between men and women of the current results (women's median value: 667, 95% normal reference interval: 241–1667) (men's median value: 623, 95% normal reference interval: 206–1733 cells/*μ*L) from the previous study done by Kassu et al. in Ethiopia (women median value: 617, range 258–1301 cells/*μ*L) (men's median value 619, reference range: 249–1,933 cells/*μ*L) from Akaki, but the upper limit of 95% normal reference interval for men (206–1733 cells/*μ*L) was completely higher than the values of men those found in Wonji (range: 180–1253 cells/*μ*L) [16]; probably it could be environmental factors (Table 4).

The absolute CD8^+^ T lymphocytes mean, median, and the 95% normal reference interval value for the current study were also greater than the studies conducted from Switzerland by Bisset et al. (median: 347, range: 137–823 cells/*μ*L) [19], Shanghai, China (mean: 539, median: 503, range: 336–780 cells/*μ*L) [24], Asian population (mean: 642, median: 616, reference range: 243–1206 cells/*μ*L) [25]; the difference could be on our apparently healthy study population whereas they used only blood donors especially in Asian population and Switzerland studies, we used more numbers of study subjects in the current study but the study done in Switzerland used 70 individual healthy blood donors, or it may be due to the high parasitic prevalence and high microbial infection in the local area which can contribute to the increased number of absolute CD8^+^ T lymphocyte counts, since once CD8^+^ T lymphocytes are activated, they will have long life span.

Absolute CD4^+^ to CD8^+^ ratio T lymphocytes mean, median, and 95% normal reference interval were lower than from women's, but the difference was not statistically significant (*P* = 0.26).

Furthermore the result of this gender specific ratio in the current study (males median: 1.23, normal reference interval: 0.4–2.8) (females median: 1.26, normal reference interval: 0.6–2.5) is in line with the study conducted Akaki (males median: 1.10, reference range: 0.41–2.57) (females median: 1.2, reference range: 0.52–2.60), and also Wonji (males median 1.4, range: 0.6–2.80), south east of Ethiopia [16].

The gender specific result CD4^+^ to CD8^+^ ratio (males mean: 1.3 ± 0.5, normal reference interval: 0.4–2.8) (females mean: 1.34 ± 0.5, normal reference interval: 0.6–2.5) of the current study was lower than from the study done in Uganda (males mean: 1.47, range: 0.54–3.94) (females mean: 1.46, range: 0.48–4.41) [8].

Overall the result of CD4 to CD8 T lymphocytes ratio in the current study was lower than the study from Kenya [20], Tanzania [10], Nigeria [11], and India [18]. As compared to the study done in Switzerland [19] and Asian population [25], the current results also show great variation (Table 5), since our life style exposed to different infectious organism and the high prevalence of infection for the current study have been implicated as possible explanation to reduce CD4/CD8 T-cell ratios.

## 5. Conclusions

The overall CD3^+^ T lymphocytes reference values were high and its interval was very wide.

The mean and 95% normal reference interval values for absolute CD4^+^ T lymphocyte strength the earlier reports done from Ethiopia and other African studies; it was low CD4^+^ T lymphocyte. In general, in this study, very high CD8^+^ T lymphocytes counts and very low CD4^+^ to CD8^+^ ratio were observed; so the current reference interval results should be applicable only for the study area. Since the reference value for CD4^+^ T lymphocyte is low in the current study from the national reference and user guidelines also, physician should use a higher CD4^+^ T lymphocytes cutoff values for initiation of ART drugs and highly recommended to establish local reference interval for each laboratory.

## Figures and Tables

**Table 1:** Sociodemographic characteristics of HIV1/2 negative adults who visited VCT in University of Gondar Hospital, Ethiopia, 2013.

Sociodemographic characteristic	Number of participants	Percent (%)
Marital status		
Single	224	70
Married	48	15
Divorced	46	15.4
Widowed	2	0.6
Occupational status		
Student	95	29.7
Farmer	37	11.6
Daily labor	36	11.3
Merchant	31	9.7
Housewife	24	7.5
Teacher	7	2.2
Driver	5	1.6
Others	85	26.6
Educational status		
Illiterates	50	15.6
1–8th	94	29.4
9–12th	116	36.3
>12th	60	18.8
Ethnic groups		
Amhara	292	91.3
Kimant	12	3.8
Oromo	3	0.9
Tigray	2	0.6
Others	11	3.4

**Table 2:** Means, medians, and 95th percentile reference intervals of CD3^+^, CD4^+^, CD8^+^, and CD4 to CD8 ratio of HIV negative adults who visited VCT, University of Gondar Hospital, Ethiopia, 2013.

Participants	Average CD3^+^/*µ*L	Absolute CD4^+^/*µ*L	Absolute CD8^+^/*µ*L	CD4 : CD8 ratio
Male = (161)				
Mean ± SD^∗^	1567 ± 562	759 ± 252	705 ± 370	1.3 ± 0.5
Median	1485	737	623	1.23
95% range	611–2974	265–1685	206–1733	0.4–2.8
Female (159)				
Mean ± SD	1706 ± 547	882 ± 275	739 ± 342	1.34 ± 0.5
Median	1675	858	667	1.26
95% range	828–2824	387–1411	241–1667	0.6–2.5
Total (320)				
Mean ± SD	1636 ± 559	820 ± 270	722 ± 356	1.3 ± 0.5
Median	1574	774	650	1.2
95% range	655–2823	321–1389	220–1664	0.5–2.52

^*∗*^SD = standard deviation.

**Table 3:** The mean reference value of T lymphocytes subpopulation/*µ*L blood in HIV negative adults who visited VCT, University of Gondar Hospital, Ethiopia by age and sex, 2013.

Age group (yrs.)	Gender											
	Male				Female				Total			
	Number tested	Mean CD4^+^	Mean CD8^+^	Mean CD4/CD8	Number tested	Mean CD4^+^	Mean CD8^+^	Mean CD4/CD8	Number tested	Mean CD4^+^	Mean CD8^+^	Mean CD4/CD8
18–25	96	791	735	1.3	122	891	740	1.4	218	847	737	1.3
26–35	46	705	662	1.3	25	884	764	1.3	71	768	698	1.3
36–45	15	761	649	1.4	10	854	721	1.4	25	798	678	1.4
46–60	2	565	876	0.6	2	485	472	1.2	4	525	674	0.9
>60	2	636	534	2	—	—	—	—	2	636	534	2.0
Total	**161**	**759**	**705**	**1.3**	**159**	**882**	**739**	**1.34**	**320**	**820**	**722**	**1.3**

**Table 4:** Gender based 95% reference interval of HIV negative adults visited VCT, University of Gondar Hospital, Ethiopia, 2013, as compared with other countries.

Parameter	Current study	Ethiopia	CAR	Nigeria	India	Switzerland
Males						
CD3^+^/*µ*L	611–2974	759–2742	NA	NA	NA	521–1772
CD4^+^/*µ*L	265–1685 355–1213^∗^	391–1145	380–1617	351–1455	383–1347	336–1126
CD8^+^/*µ*L	206–1733	249–1933	267–1545	155–863	NA	125–780
CD4 : CD8 ratio/*µ*L	0.4–2.8	0.41–2.57	0.34–1.88	0.7–5.1	NA	0.9–6.0

Females						
CD3^+^/*µ*L	828–2824	741–2329	NA	NA	NA	595–1861
CD4^+^/*µ*L	387–1411 470–1298^∗^	386–1355	386–1454	383–1654	448–1593	314–1270
CD8^+^/*µ*L	241–1667	258–1301	226–1225	133–919	NA	147–836
CD4 : CD8 ratio/*µ*L	0.6–2.50	0.52–2.60	0.6–2.27	0.8–5.8	NA	1.0–4.9
References in numbers		[16]	[17]	[11]	[18]	[19]

^*∗*^User's guideline values, NA = not available.

**Table 5:** The overall 95% normal reference interval for Tlymphocytes in HIV negative adults in the current study as compared to other African countries.

Parameter	User's guideline	Current study	Previous study in Ethiopia	Kenya	Tanzania	Uganda	Nigeria
CD3^+^/*µ*L	688–1955	655–2823	NA	744–2634	NA	NA	NA
CD4^+^/*µ*L	500–1300	321–1389	366–1235	421–1550	312–1367	418–2105	365–1571
CD8^+^/*µ*L	208–796	220–1664	311–1618	210–1081	168–996	256–1619	145–884
CD4 : CD8 ratio/*µ*L	0.83–6.10	0.5–2.5	0.4–2.4	0.9–3.3	1.1–2.5	0.52–4.1	0.5–5.3
References in numbers			[14]	[20]	[10]	[8]	[11]

NA = not available.
